# Low-Frequency Intrapulmonary Percussive Ventilation Increases Aerosol Penetration in a 2-Compartment Physical Model of Fibrotic Lung Disease

**DOI:** 10.3389/fbioe.2020.01022

**Published:** 2020-08-28

**Authors:** Sandrine Le Guellec, Laurine Allimonnier, Nathalie Heuzé-Vourc’h, Maria Cabrera, Frédéric Ossant, Jérémie Pourchez, Laurent Vecellio, Laurent Plantier

**Affiliations:** ^1^INSERM, Research Center for Respiratory Diseases, U1100, Tours, France; ^2^DTF Aerodrug, Tours, France; ^3^Université de Tours, Tours, France; ^4^Inserm U1253, Imagerie et Cerveau, Tours, France; ^5^Mines Saint-Etienne, Univ. Lyon, Univ. Jean Monnet, INSERM, U1059 Sainbiose, Centre CIS, Saint-Etienne, France; ^6^CHRU de Tours, Service de Pneumologie et Explorations Fonctionnelles Respiratoires, Tours, France

**Keywords:** pulmonary fibrosis, nebulization, physiology, compliance, aerosol, inhalation

## Abstract

In patients with fibrotic pulmonary disease such as idiopathic pulmonary fibrosis (IPF), inhaled aerosols deposit mostly in the less affected region of the lungs, resulting in suboptimal pharmacokinetics of airway-delivered treatments. Refinement of aerosol delivery technique requires new models to simulate the major alterations of lung physiology associated with IPF, i.e., heterogeneously reduced lung compliance and increased airway caliber. A novel physical model of the respiratory system was constructed to simulate aerosol drug delivery in spontaneously breathing (negative pressure ventilation) IPF patients. The model comprises upper (Alberta ideal throat) and lower airway (plastic tubing) models and branches into two compartments (Michigan lung models) which differ in compliance and caliber of conducting airway. The model was able to reproduce the heterogeneous, compliance-dependent reduction in ventilation and aerosol penetration (using NaF as a model aerosol) seen in fibrotic lung regions in IPF. Of note, intrapulmonary percussive ventilation induced a 2–3-fold increase in aerosol penetration in the low-compliance/high airway caliber compartment of the model, demonstrating the responsiveness of the model to therapeutic intervention.

## Introduction

Fibrotic interstitial lung diseases (ILD) are a group of severe chronic non-communicable lung diseases where excessive deposition of abnormal extracellular matrix, in association with epithelial and/or endothelial lesions, results in progressive respiratory failure. Idiopathic pulmonary fibrosis (IPF) is the most common and the most severe idiopathic ILD (King et al., 2011). In IPF, fibrotic lesions follow the heterogeneous “usual interstitial pneumonia” pattern where fibrotic lesions alternate with preserved regions, and predominate in peripheral (supleural) areas.

Current treatments for IPF are unsatisfactory. Although two therapies, pirfenidone and nintedanib, both slow the decline of lung function (Noble et al., 2011; King et al., 2014; Richeldi et al., 2014) and may increase survival (Canestaro et al., 2016), neither drug blocks or reverses the progress of disease and their tolerance is fair at best (Fletcher et al., 2016). There is a need for new drugs and treatment delivery routes for IPF and other ILDs.

IPF is spatially restricted to the lung, making it an ideal candidate disease for the use of topical, i.e., inhaled drugs. Aerosolized delivery through the airways has key advantages over the oral or injected routes for the administration of drugs to the lungs. Aerosolization is non-invasive and provides high therapeutic index. Aerosolized drug delivery is used routinely in patients with airway diseases. By contrast, the inhaled route is not currently used for the treatment of IPF, owing in part to low aerosol deposition in the lung.

IPF is associated with multiple alterations in lung structure with potential to hinder penetration of inhaled aerosols (Plantier et al., 2018). Fibrosis leads to major reductions in lung compliance (Zielonka et al., 2010) which are presumed heterogeneous given the spatial heterogeneity of lesions, and which result in reduced ventilation of the affected regions. Lung compliance is defined by the ratio of change in lung volume (V) over change in the transpulmonary pressure (Ptp). Besides fibrosis of alveolar regions, bronchial lesions may be observed whose nature is debated. Although the total number of airways in reduced in end-stage IPF (Verleden et al., 2020), the number of visible distal airways in increased in lungs with severe IPF (Verleden et al., 2020), while conducting airway volume is increased by 32% in patients with moderate IPF in comparison with control subjects (Plantier et al., 2016) and expiratory airflow is increased (Plantier et al., 2018).

Anatomical and physiological alterations of the respiratory system in IPF may result in altered deposition of inhaled aerosols. Similar total lung deposition, as measured by concentrations in bronchoalveolar lavage fluid (Khoo et al., 2020) or planar scintigraphy (Samuel and Smaldone, 2019), can be achieved in IPF patients as compared to healthy subjects. However, planar scintigraphy images suggest that deposition of micrometric aerosols is not homogenous in some IPF patients, in contrast with healthy subjects (Kanazawa et al., 1993; Khoo et al., 2020). Similarly, deposition of the nanometric aerosol Technegas was patchy and predominated in the upper regions of the lungs, which are typically the less damaged areas in 2 IPF patients (Watanabe et al., 1995). We hypothesize that reduced lung compliance results in reduced penetration and subsequent heterogeneous deposition of inhaled aerosols in IPF.

At present, inhalation devices and protocols allow high aerosol penetration and deposition in ventilated regions, but poor penetration and deposition in the regions of the respiratory system receiving little or no ventilation. The preferential deposition of aerosols in the less damaged areas needs to be overcome for satisfactory spatial targeting of inhaled therapeutics in IPF. It is therefore important to develop optimized aerosolization protocols allowing to target the low compliance alveolar regions in IPF. Models able to simulate IPF lungs under spontaneous ventilation are required to test the efficacy of such protocols.

Acoustic/pulse pressure waves hold potential to increase aerosol delivery in low compliance lungs. Indeed, acoustic waves increase aerosol penetration and deposition in the maxillary sinuses (Durand et al., 2012; Möller et al., 2014; Leclerc et al., 2015; Moghadam et al., 2018). Maxillary sinuses are bone structures, which have near-null compliance and thus receive no ventilation during the respiratory cycle. Multiple experiments established that acoustic waves were most effective in the 100–300 Hz frequency range, close to the resonance frequency of the maxillary sinuses (Durand et al., 2012; Möller et al., 2014; Leclerc et al., 2015; Moghadam et al., 2018).

In light of these elements, we hypothesized (1) that poor penetration of inhaled aerosols in low compliance lung regions could be reproduced in a two-compartment mechanical model of fibrotic lungs under negative-pressure (spontaneous) ventilation and (2) that acoustic pressure waves near the resonance frequency of the model would increase aerosol penetration in the low compliance compartment.

## Materials and Methods

### Construction of a Two-Compartment Model of the Respiratory System

**FIGURE 1:**
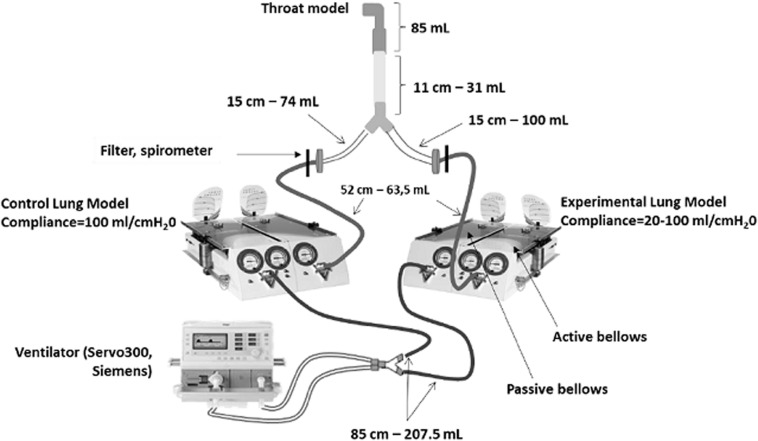
Schematic of the two-compartment model of the respiratory system used to simulate IPF pathophysiology. The length and volume (measured) of corrugated plastic tubing used to model the airways are featured.

A two-compartment model of the entire respiratory system (schematic in Figure 1) was constructed. This model comprised a physical model of the upper airways (Alberta ldealized throat, Copley Scientific, United Kingdom), a plastic tube modeling the subglottic airways down to the 4th bronchial division, and a Y-piece connecting each lung compartment. Each lung compartment included a plastic tube modeling the distal airways, and a mechanical lung model (Michigan Lung Model, Michigan Instruments, United States) modeling the alveolar regions. Each mechanical lung model comprised (1) a first bellows connected to the airway model described above (“active bellows”) and (2) a second bellows (“passive bellows”), which was mechanically coupled to the first bellows, and was ventilated using a clinical ventilator (Servo 300, Siemens, Germany). Thus, the first bellows was ventilated under negative pressure, allowing to simulate physiological spontaneous breathing. Airway diameter and bellows compliance could be individually adjusted. To simulate normal lung, in the “Control compartment” airway diameter was 17 mm, and lung compliance was 100 ml/cmH_2_O. To simulate fibrotic lung, in the “Experimental compartment” airway diameter was 22 mm, and lung compliance was ≤ 100 ml/cmH_2_O. Overall, the volumes corresponded to those observed in an adult man. Filters (Anest-Guard, Teleflex, France) were inserted in each compartment, at the junction of the tube modeling the airways and the bellows modeling the alveolar regions.

### Distribution of Ventilation in the Model

For characterization of ventilation distribution, the model was actuated under the following ventilator settings: Frequency = 10/min, inspiratory pressure between 5 and 18 cmH_2_O, inspiratory time = 2 s. These settings simulate spontaneous breathing in an adult subject. Ventilation of each compartment was measured with spirometers (300 L/min, AD Instruments, Dunedin, New Zealand) inserted in place of the filters (Figure 1). In addition, the resonance frequency of the model were measured using a clinical forced oscillations device (Tremoflo, EMKA, France).

### NaF Aerosol Delivery and Quantitation of Aerosol Deposition

The model was connected to a jet nebulizer aerosol generator (Atomisor AOHBOX/NL9M, DTF medical, France) at the upper airway opening. This generator delivers an aerosol with 4–5 μm mass median aerodynamic diameter. Inspiratory and expiratory valves were used. For all aerosol delivery experiments, the pulmonary model was actuated with the following ventilator settings to simulate tidal ventilation: Frequency = 10/min, inspiratory time = 2 sec, inspiratory pressure = 5 cmH_2_O. Three milliliter of a 2.5% sodium fluoride (NaF) solution were nebulized over 10–15 min. After each nebulization session, the filters were dismounted. The filters were flushed with 30 ml of 2% vol./vol. aqueous total ionic strength adjustment buffer (TISAB) solution (Sigma-Aldrich, St. Louis, MO, United States) and incubated for 3 h at room temperature. NaF was then assayed by electrochemical analysis (SevenGo Pro, Mettler Toledo, United States). The quantity of NaF aerosol deposited in each filter (Control or Experimental) was expressed as a fraction of the initial NaF dose that was loaded in the nebulizer.

### Aerosol Distribution in the Model

Aerosol distribution was controlled for each nebulization experiments by dosing all parts of the model. Aerosol deposition was quantitated at the level of the nebulizer chamber, expiratory filter, construct, upper airway (Alberta throat model, trachea tube and Y-piece), and each lung compartment (tubing and filter). Each section was washed with 30–250 ml TISAB and NaF was assayed as described above.

### Delivery of Acoustic/Pulse Pressure Waves

**FIGURE 2:**
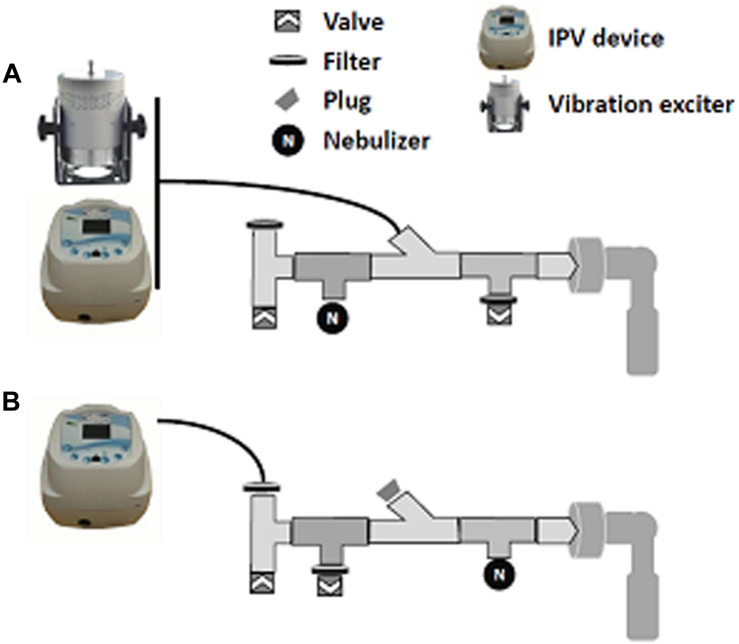
Constructs used for acoustic/pulse pressure wave delivery during nebulization. All constructs are connected to the throat model (right). **(A)** Construct used for acoustic wave delivery and initial IPV delivery. **(B)** Construct used for multiple-frequency IPV delivery.

To generate acoustic pressure waves in the 20–500 Hz frequency range, sinusoidal signals were generated with VB Generator freeware^1^, amplified (t.amp PM40C, Thomann, Germany), and transmitted to a vibration exciter (S-50009, Tira GmbH, Germany). Amplifier gain was set so that acoustic pressure was identical across the frequency range, using a sonometer; 80 dB acoustic waves could be generated at all frequencies. To generate pulse pressure waves in the 1–10 Hz frequency range, a Pegaso (Dima Italia, Italy) intrapulmonary percussion ventilation device was used. IPV devices deliver small volumes of air; the shape of the signal is not sinusoidal (Toussaint et al., 2012). IPV pulse pressure was set at 40 cmH_2_O and the inspiratory/expiratory ration was set to 1. In a first set of experiments, the vibration exciter and the IPV device were connected to the nebulizer/valves construct as shown in Figure 2A. In a second set of experiments, the IPV device was connected as shown In Figure 2B following optimization experiments. Both high-frequency acoustic waves and low-frequency IPV pulses were delivered to the model for the duration of nebulization.

### Statistical Analysis

All data were expressed as means and standard deviations. Group by group comparisons were performed with Student’s *t*-test (2 groups) or ANOVA (more than 2 groups). Paired or unpaired analyses were done as appropriate. Relationships between variables were assessed by linear regression. Prism v5.0 (Graphpad, San Diego, CA, United States) was used for all analyses.

## Results

### Ventilation and Aerosol Deposition in the Model

Firstly, distribution of ventilation (tidal volume) between the two compartments of the models was assessed. As expected, tidal volume in both compartments of the model was a linear function of the pressure setting on the ventilator, which was applied to the passive bellows of the lung model, and of the compliance setting of the lung model (Supplementary Figure S1). For a given level of ventilation pressure and compliance, tidal volume was not significantly different between compartments.

**FIGURE 3:**
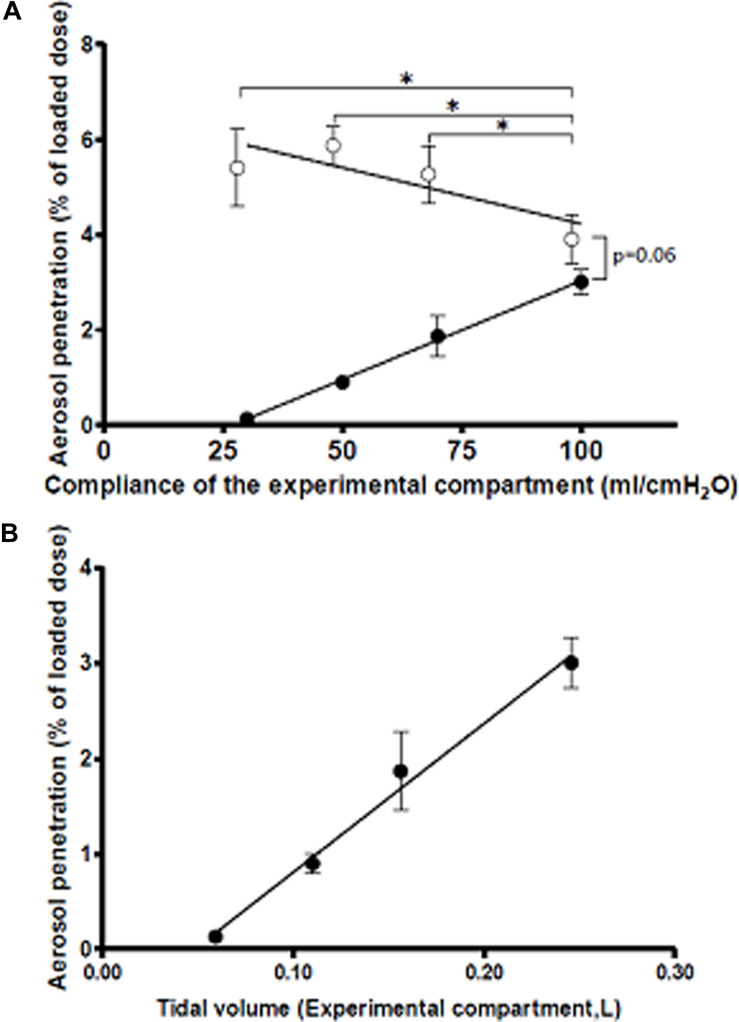
Aerosol deposition in the Control (open circles) and Experimental (filled circles) as a function of **(A)** compliance of the experimental compartment and **(B)** tidal volume in the experimental compartment. Compliance was set at 100 ml/cmH_2_O in the Control compartment). *p < 0.05. Regression lines are shown. *N* = 3. Means and SD.

Then, a NaF aerosol was delivered into the model while ventilated, and deposition onto the compartment filters, which assesses aerosol penetration into the respective compartment, was measured. When the compliance of both compartments was set at 100 ml/cmH_2_O, aerosol deposition in the Experimental compartment (then characterized only by 33% larger airway) trended to be slightly reduced in comparison with the Control compartment of the model (3.9 ± 0.5% of the loaded dose vs. 3.0 ± 0.3%, *p* = 0.06 by paired analysis – Figure 3A).

Then, aerosolization experiments were repeated when compliance of the Experimental compartment was decreased to 70, 50, and 30 ml/cmH_2_O. As shown in Figure 3A, decreasing compliance of the Experimental compartment had two effects. First, an increase in aerosol penetration was observed in the Control compartment; this increase correlated negatively with compliance in the Experimental compartment (*R*^2^ = 0.48, *p* = 0.012). Second, aerosol penetration decreased in the Experimental condition; this decrease followed a strong linear relationship with compliance (*R*^2^ = 0.96, *p* < 0.0001). Thus, these experiments demonstrated that decreasing lung compliance in one compartment of the system shifted penetration of an inhaled aerosol away from the low compliance compartment and into the compliant compartment. As shown in Figure 1B, aerosol penetration in the Experimental compartment was strongly (*R*^2^ = 0.95, *p* < 0.0001) associated with tidal volume, supporting the hypothesis that regional reduction in lung compliance and subsequent reduction in local ventilation are key drivers in reducing aerosol deposition in fibrotic lungs.

For further experiments, the compliance of the Experimental compartment was set at 50 ml/cmH_2_O, so that the model could detect both increases and decreases in aerosol penetration following delivery of acoustic/pulse pressure waves. Oscillometry parameters were determined under this setting. Under these conditions, the resonance frequency of the system was 5 Hz.

### 20–500 Hz Acoustic Waves Failed to Increase Aerosol Penetration in the Low Compliance Compartment

**FIGURE 4:**
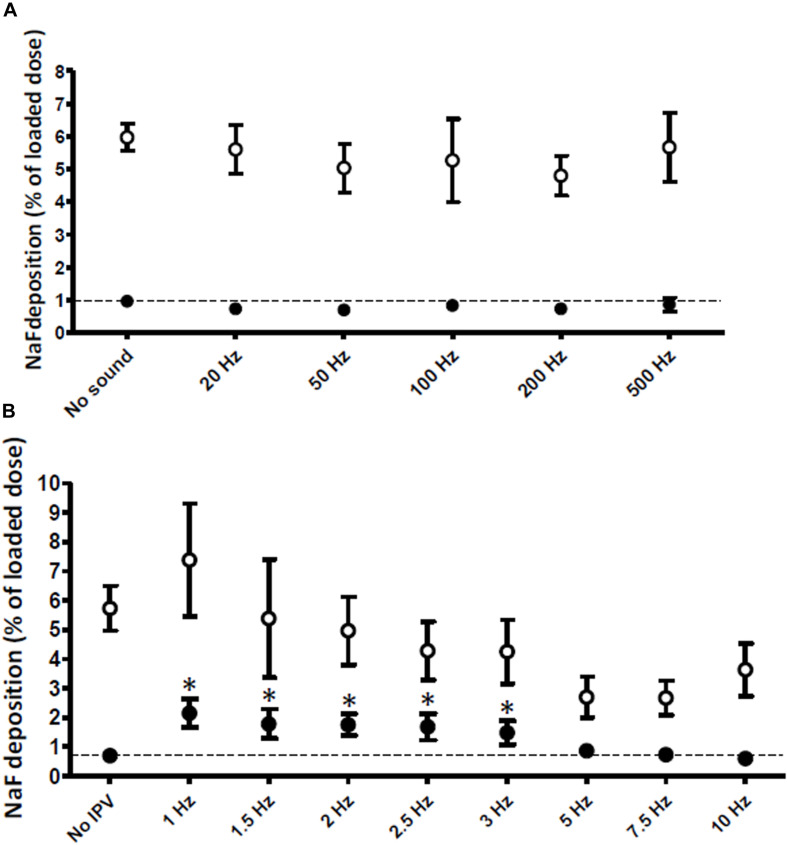
Aerosol deposition in the Control (100 ml/cmH_2_O compliance, open circles) and Experimental (50 ml/cmH_2_O compliance, filled circles) compartments of the model. **(A)** 20–500 Hz audible acoustic waves (*n* = 3) and **(B)** 1–10 Hz IPV pressure pulses (*n* = 6) were delivered during nebulization. Dotted lines show the control condition (no sound or no IPV). *p < 0.05 vs. no IPV. Means and SD.

Audible acoustic waves are known to increase aerosol penetration in the maxillary sinuses (Leclerc et al., 2015). Thus, in a first set of experiments, 80 dB/20–500 Hz acoustic waves were delivered to the nebulizer construct for the duration of nebulization using the construct shown in Figure 2A. As shown in Figure 4A, audible acoustic waves had no effect on aerosol penetration either in the Control or in the Experimental compartment, and the Experimental/control deposition ratio was unchanged.

In maxillary sinuses, matching acoustic wave frequency with the resonance frequency of the target structures results in increased aerosol deposition (Durand et al., 2012). Because the measured resonance frequency of the model (5 Hz) was much lower than 20 Hz, we raised the hypothesis that low-frequency pressure waves were required to increase aerosol deposition. To deliver such pressure waves, an intra-pulmonary percussion ventilation (IPV) device was used. IPV devices offer important advantages in the context of aerosol deposition in ILD as (1) they are already certified for clinical use in humans including in association with inhaled aerosols, and (2) they have been shown to increase aerosol penetration in a simple one-compartment model independent of compliance (Karashima et al., 2019).

### Low-Frequency IPV Pressure Waves Increase Aerosol Penetration Twofold in the Low Compliance Compartment

Initial experiments using IPV (3 Hz, 40 cmH_2_O) delivered through the same nebulizer construct as the one used for audible acoustic wave delivery showed, in comparison with absence of pulse pressure waves, (1) that aerosol deposition in both the Control and Experimental compartments was decreased but (2) that the ratio of aerosol deposited in the Experimental/Control compartments increased from 0.16 to 0.41 (Supplementary Figure S2). Additional experiments were then conducted to evaluate the effect of IPV (3 Hz) delivered through different nebulizer constructs (Supplementary Figure S3). As shown in Supplementary Figure S4A, aerosol deposition in the Experimental compartment was increased by IPV (3 Hz) in 2 out of 6 constructs. To verify that the increase in aerosol penetration was IPV-dependent and not only construct-dependent, aerosol penetration was measured in constructs 3 and 4 both with and without IPV (3 Hz). IPV increased aerosol penetration in the Experimental compartment in both constructs (Supplementary Figure S4B).

Construct 3 was retained for further experiments where the effect of IPV frequency on aerosol penetration was tested. As shown in Figure 4B, low-frequency IPV pressure waves in the 1–3 Hz frequency range increased aerosol penetration in the Experimental compartment. The increase was maximal (twofold) for 1 Hz IPV. By contrast, 10 Hz IPV decreased aerosol penetration in the Experimental compartment. IPV did not significantly increase aerosol penetration in the Control compartment of the model although a trend was present (+29%, *p* = 0.08).

**FIGURE 5:**
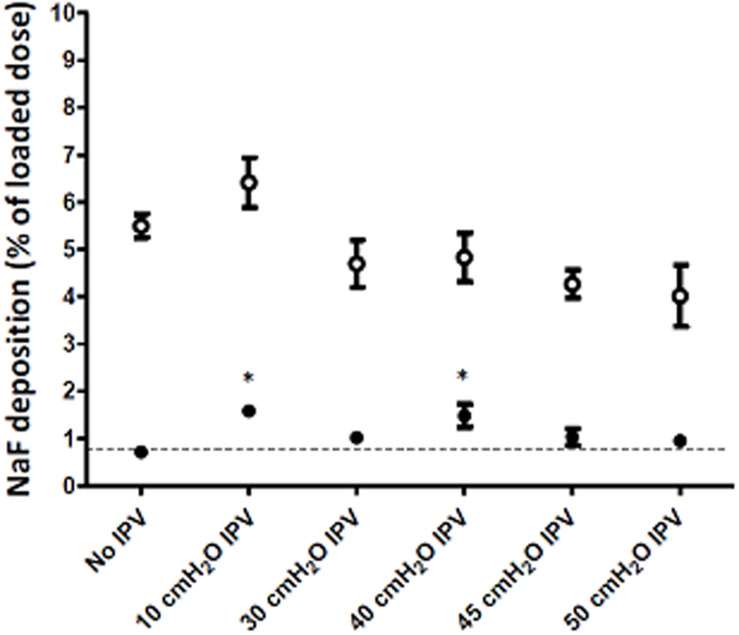
Aerosol deposition in the Control (100 ml/cmH_2_O compliance, open circles) and Experimental (50 ml/cmH_2_O compliance, filled circles) compartments of the model. Aerosol was delivered with either no IPV or 1 Hz IPV at different pressure levels. *p < 0.05 vs. no IPV.

Because low-frequency, high pressure IPV may be uncomfortable in man, an additional experiment was conducted using low-pressure (10 cmH_2_O) IPV. As shown in Figure 5, 1 Hz, 10 cmH_2_O IPV increased aerosol penetration in the Experimental compartment by 121% in comparison with no IPV.

### Aerosol Distribution in the Model

In the normal lung model, 97.8 ± 12.1% of aerosol was recovered (Table 1). 12 ± 1.7% of aerosol deposited in the respiratory system (including upper airways and lung models) while 85.8 ± 10.6% were recovered outside the model (including nebulizer, expiratory filter and construct). When compliance was reduced in the experimental compartment to simulate IPF, 90.7 ± 7.8% was recovered owing to reduced deposition in both lung and delivery apparatus. Under 1 Hz–40 cmH_2_O IPV conditions, 95.5 ± 17.2% of total aerosol was recovered. In addition to a threefold increase of aerosol deposition in the experimental compartment’s filter, IPV induced increases in the construct and the throat model, as well as a ∼50% decrease in the expiratory filter.

**TABLE 1:** Mass balance of aerosol deposition experiments.

	Normal lung model (*n* = 3)	Fibrotic lung model, no IPV (*n* = 6)		Fibrotic lung model with 1 Hz–40 cmH_2_O IPV (*n* = 6)	
Deposition site	Deposition	Deposition	*p*-value vs. Normal	Deposition	*p*-value vs. Fibrotic
Expiratory filter	27.9 ± 6.7%	32.2 ± 4.4%	0.651	17.8 ± 5.3%	**0.001**
Construct	7.1 ± 1.6%	4.8 ± 1.5%	0.530	14.1 ± 4.2%	**0.009**
Nebulizer	50.7 ± 8.0%	45.5 ± 5.9%	0.896	49.1 ± 7.8%	0.509
Alberta throat	1.7 ± 0.3%	0.9 ± 0.3%	**0.009**	4.0 ± 1.1%	**0.002**
Trachea and Y-piece	0.6 ± 0.3%	0.6 ± 0.3%	0.910	0.5 ± 0.1%	0.352
Control bronchus	0.4 ± 0.3%	0.4 ± 0.1%	0.580	0.3 ± 0.2%	0.253
Control compartment	4.6 ± 1.2%	5.5 ± 0.6%	0.578	7.4 ± 1.9%	1.000
Experimental bronchus	0.4 ± 0.3%	0.2 ± 0.1%	0.219	0.2 ± 0.2%	0.079
Experimental compartment	4.4 ± 1.6%	0.7 ± 0.2%	**0.020**	2.2 ± 0.5%	**0.002**
Total	97.8 ± 12.1%	90.7 ± 7.8%	0.899	95.5 ± 17.2%	0.626

**Deposition in the normal lung model (compliance of both compartments set at 100 ml/cmH_2_O) was compared with the fibrotic lung model (compliance of the Experimental compartment set at 50 ml/cmH_2_O) without IPV. Deposition in the fibrotic model with 1 Hz–40 cmH_2_O IPV was compared with the fibrotic lung model without IPV. Aerosol was expressed as % of the dose loaded in the nebulizer. *p* values < 0.05 were shown in bold face.**

## Discussion

In this study, we developed a novel 2-compartment model of the respiratory system simulating fibrotic lung disease, and we showed that heterogeneous reductions in lung compliance resulted in (1) an increase of aerosol penetration in the preserved lung regions and (2) proportionate decreases in tidal volume and aerosol penetration in the less-compliant region. The strength of the associations between lung compliance, tidal volume and aerosol penetration support the hypothesis that reduced lung compliance is the main determinant of reduced aerosol deposition in fibrotic lung regions. Then, we used this model to explore whether acoustic/pulse pressure waves modulated aerosol penetration, and showed that low-frequency intrapulmonary pressure ventilation pulses reliably increased aerosol penetration in the low compliance compartment.

Although the total amount of aerosol-delivered drugs may be similar in patients with fibrotic lung disease and healthy controls at the level of the whole organ (Diaz et al., 2012; Khoo et al., 2020), imaging studies suggest that inhaled aerosols may mainly deposit into the less-affected regions of the lungs and thus presumably miss their cellular targets (Kanazawa et al., 1993; Watanabe et al., 1995). Despite recent demonstration that small aerosol particles (1.5 μm diameter) and polydisperse aerosols should be preferred for IPF patients (“The Topical Study of Inhaled Drug (Salbutamol), 2019 Delivery in Idiopathic Pulmonary Fibrosis. – PubMed – NCBI” n.d.), preferential deposition of aerosols in the less damaged regions needs to be overcome. Optimizing spatial targeting is a major challenge to develop inhaled therapies for diseases which, like IPF, are characterized by heterogeneously distributed fibrotic lung lesions.

Experimental models are required to understand the mechanisms driving aerosol penetration and deposition in fibrotic lung disease. To this day, few studies were conducted in the aim of simulating both disease mechanics and aerosol deposition in fibrotic lungs under spontaneous ventilation. Montigaud et al. (2019), developed an *ex vivo* model combining heat-treated porcine lungs and a 3D-printed replica of the human airways. Although this model better simulates a branched airway anatomy, allows to replicate reductions in lung compliance predominating in subpleural regions, and can be used for aerosol deposition studies, it is unwieldy and cannot be reused for multiple experiments. To our knowledge, the mechanical model described herein is the first to offer the possibility to simulate the geometrical and physical characteristics of either mild or severe, restrictive or obstructive (using airflow resistances) lung disease *in vitro*, and to conduct replicated experiments of inhaled drug delivery. The amount of nebulized aerosol that deposited in lower airways of the normal lung model (trachea, Y-piece, bronchi and lung compartments) was approximately 10%, which is similar to observations in healthy volunteers and patients with lung disease during inhalation experiments with jet nebulizer devices (Rau, 2005; Dugernier et al., 2017). This suggests that this new *in vitro* model does simulate *in vivo* aerosol deposition. The main limitation of the mechanical model lies in the fact that it is not clear whether a separate low compliance lung compartment would act the same as isolated low compliance hotspots embedded in otherwise normal lung parenchyma.

Our experiments support that heterogeneous reductions in lung compliance could play a role in the distribution of aerosol penetration in fibrotic lung disease. Indeed, we observed both increased aerosol penetration in the normal-compliance compartment, and reduced aerosol penetration in the reduced-compliance compartment. Although the clinical relevance of this finding remains to be defined, preferential deposition of inhaled drugs to preserved lung regions may result in a reduced therapeutic index and a requirement for higher doses. There is thus a need for inhaled drug delivery methods allowing to increase aerosol penetration and deposition in fibrotic, low compliance lung regions.

The requirement to deliver drugs to low compliance regions of the respiratory system led to the discovery that audible acoustic waves enhance deposition of nasally-delivered aerosolized drugs into maxillary sinuses (Möller et al., 2014). By analogy, we hypothesized that acoustic/pressure waves may increase aerosol penetration in the fibrotic lung model. Interestingly, although audible acoustic waves had no effect, contrary to sinus models, low-frequency IPV pulses had a marked effect and increased aerosol penetration threefold. Although the high variance in total mass balance mandates caution in the interpretation of aerosol deposition experiments, the effect of IPV on aerosol deposition in the experimental compartment was highly reproducible. This result is in line with the observation by others that IPV increases penetration of therapeutic aerosols in a single compartment mechanical model of the respiratory system, independent of model compliance (Karashima et al., 2019).

The mechanisms by which pressure pulses, such as those delivered by IPV, increase aerosol penetration remain unknown. These mechanisms can be discussed by analogy with acoustic wave-enhanced delivery of aerosols from the nose to maxillary sinuses, which, like distal airspaces in fibrotic lungs, can be considered a low compliance cavity. A hypothesis is periodic compression of air by acoustic waves, generating bidirectional airflow (Xi et al., 2017). Under this hypothesis, a maximal effect is expected when transmission of the input pressure to the target cavity is maximal, that is, when the frequency of acoustic waves equals the resonance frequency of the target cavity. Indeed, matching soundwave frequency to resonance frequency of the maxillary sinuses (estimated with the Helmhotz resonator model) increases aerosol delivery (Durand et al., 2012; Leclerc et al., 2015), while frequency sweeps bracketing the estimated resonance frequency of the sinus yield optimal results (Moghadam et al., 2018). At variance with the hypothesis that acoustic waves emitted at the resonance frequency of the target may increase aerosol penetration, we observed that audible acoustic waves had no effect. Rather, increased aerosol penetration was observed using low-frequency (≤3 Hz) IPV. Whether it is important that acoustic/pressure pulse frequency is inferior to the resonance frequency of the system (here, 5 Hz) remains to be explored. Interestingly, in a 2-bottle physical model, maximal aerosol penetration aerosol is observed with 100 Hz acoustic waves, while the estimated resonance frequency of the model cavity is 303 Hz (Xi et al., 2017). The mechanisms contributing to IPV-increased aerosol penetration thus remain to fully elucidate. Acoustic radiation pressure, which is well described in the ultrasonic frequency range (Foresti et al., 2013) and is also physically relevant for low-frequency (trending toward 0 Hz) acoustic waves (Löfstedt and Putterman, 1991), may be a candidate mechanism. Importantly, it is unclear how the frequency of the pressure waves that dissipate in the airway relates to the frequency IPV volume/pressure pulses.

There is currently great interest in the development of inhaled therapies for IPF and other ILD. Inhaled antifibrotic treatments would represent a key advance in IPF. Although several inhaled drugs were or are currently being evaluated in clinical trials in IPF, such as glutathione (Borok et al., 1991), heparin (Markart et al., 2010), interferon-γ (Diaz et al., 2012), and pirfenidone (Khoo et al., 2020), none has been successful so far.

IPV is a respiratory physiotherapy technique that consists in delivery of small volumes of air into the airways, typically in the 1–10 Hz frequency and 10–50 cmH_2_O pressure ranges (Reychler et al., 2018). It is currently used to mobilize secretions, promote lung recruitment and improve gas exchange in chronic conditions such as neuromuscular diseases, cystic fibrosis, and chronic obstructive pulmonary disease. Our observation that low frequency (≤3 Hz) pressure pulses, but not high frequency (>3 Hz) pressure pulses improve aerosol deposition are in line with previous studies. Initial studies into IPV-coupled nebulized delivery in humans were disappointing (Reychler et al., 2004, 2006), although IPV devices were not the same as the one used in our study. In these studies, IPV was delivered at a frequency of 4.2 Hz, higher than the 3 Hz threshold identified in our work. Likewise, bench experiments showed that high-frequency oscillation ventilation (exact frequency not reported) results in reduced lung deposition in comparison with conventional delivery under spontaneous ventilation (Li et al., 2020). Our results suggest that IPV may be repurposed to improve aerosolized drug delivery in the clinic.

Whether low-frequency (≤3 Hz) IPV is a feasible option to improve aerosol delivery to patients with IPF depends on a number of issues, including (1) whether it does improve aerosol delivery to low compliance lung regions *in vivo*, (2) whether it can be tolerated by IPF patients, and (3) whether it is innocuous. Although IPV appears to be well tolerated at frequencies in the order of 4–6 Hz (Nava et al., 2006; Paneroni et al., 2011), whether low-frequency is tolerated for the duration required for nebulized drug delivery remains to explore. Since patient discomfort is associated with IPV pressure, our observation that 10 cmH_2_O did increase aerosol penetration in the low-compliance compartment suggests that this method may be usable in patients. Although IPV appears to be generally safe (Paneroni et al., 2011), it is essential to assess whether IPV-associated volutrauma or barotrauma is deleterious in IPF patients. Notably, whether IPV can induce IPF exacerbations will need to be ruled out before clinical application. IPF exacerbations are life-threatening events which can be triggered by medical procedures such as mechanical ventilation and lung surgery (Ryerson et al., 2015). Adverse effects of VPIP are rare in healthy subjects and patients with obstructive lung disease, with cases of pneumothorax and proximal airway obstruction attributed to handling error (Flurin et al., 2009).

## Conclusion

Using a mechanical model of fibrotic lung disease, we observed that reduced compliance of target lung regions is a critical determinant of aerosol penetration, and thus presumably of aerosol deposition. This finding underscores the need for techniques specifically tailored to enhance delivery of inhaled aerosols to low compliance regions of the lungs, if fibrotic lung disease is to be successfully treated with inhaled therapies. Low-frequency (≤3 Hz) IPV pulses may represent an attractive option to increase aerosol deposition in fibrotic lung regions.

## Data Availability Statement

The raw data supporting the conclusions of this article will be made available by the authors, without undue reservation, to any qualified researcher.

## Author Contributions

LA, SL, and MC conducted the experiments and analyzed the results. LV, FO, SL, NH-V, and JP provided scientific input into acoustic and pulse wave physics and contributed to the experiment design. LP coordinated the overall project, designed and analyzed experiments, and drafted the manuscript. All authors reviewed and approved the manuscript.

## Conflict of Interest

SL is an employee of DTF medical. LV is currently an employee of Nemera. The remaining authors declare that the research was conducted in the absence of any commercial or financial relationships that could be construed as a potential conflict of interest. The handling editor declared a past co-authorship with one of the authors NH-V.

## Footnotes

1 VB Audio. 2002. France. http://vincent.burel.free.fr/download/index.htm

## Supplementary Material

FIGURE S1 Tidal volume in the active bellows, as a function of inspiratory pressure in the passive bellows (as set on the driving respirator) and set compliance. IPV was not used for these measurements. Means of 5 measurements are shown. Standard deviations were close to zero and are not shown.

FIGURE S2 **(A)** Aerosol deposition in the Control (100 ml/cmH_2_O compliance, open circles) and Experimental (50 ml/cmH_2_O compliance, filled circles) compartments of the model. **(B)** Ratio of aerosol deposition in the Experimental and Control compartment. 3 Hz IPV was delivered using the nebulizer construct shown in Figure 2A. ^∗^*p* < 0.05.

FIGURE S3 Nebulizer construct tested for IPV-enhancer aerosol deposition experiments.

FIGURE S4 Aerosol deposition in the Control (100 ml/cmH_2_O compliance, open circles) and Experimental (50 ml/cmH_2_O compliance, filled circles) compartments of the model. **(A)** IPV (3 Hz, 40 cmH_2_O) was delivered during nebulization, using the 5 constructs shown in Supplementary Figure S3. **(B)** Constructs 3 and 4 were used either without or with IPV (3 Hz, 40 cmH_2_O).
