# Supplementary figures and images for: Low-Frequency Intrapulmonary Percussive Ventilation Increases Aerosol Penetration in a 2-Compartment Physical Model of Fibrotic Lung Disease
Source: Front Bioeng Biotechnol. 2020 Aug 28;8:1022. doi: 10.3389/fbioe.2020.01022 (PMC7483496; doi:10.3389/fbioe.2020.01022)

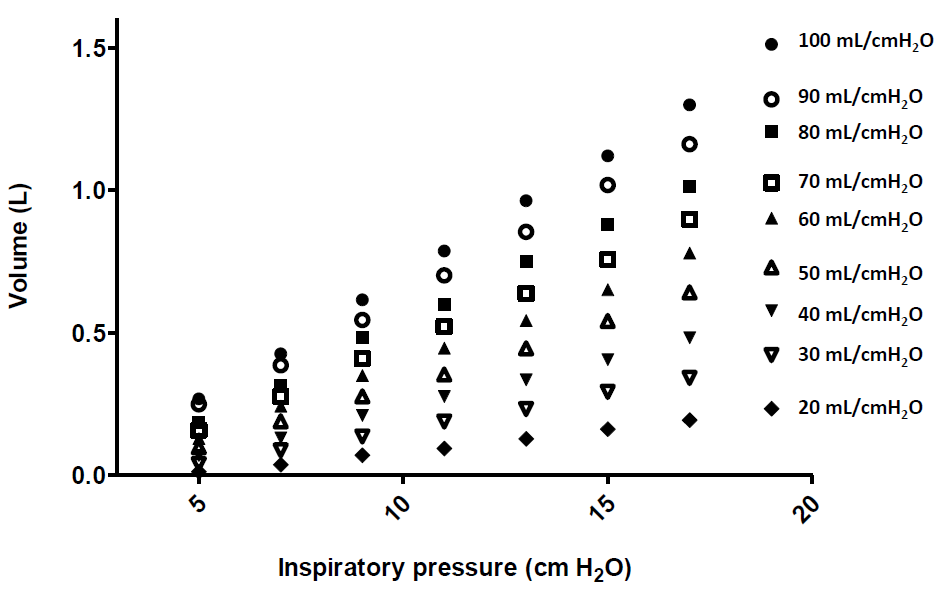

Supplement: FIGURE S1 — Tidal volume in the active bellows, as a function of inspiratory pressure in the passive bellows (as set on the driving respirator) and set compliance. IPV was not used for these measurements. Means of 5 measurements are shown. Standard deviations were close to zero and are not shown. [file Image_1.tif]

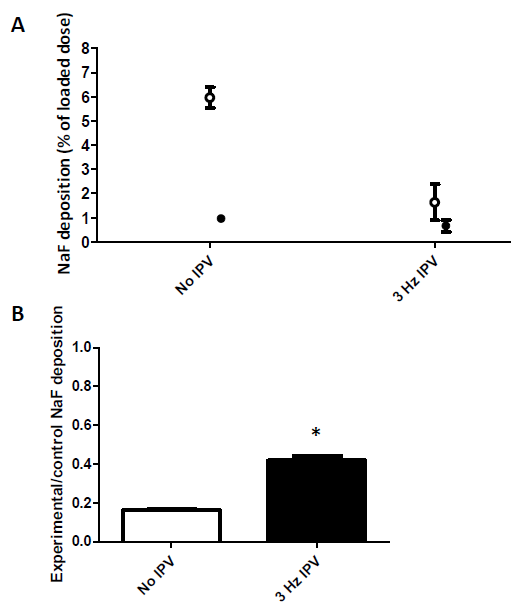

Supplement: FIGURE S2 — (A) Aerosol deposition in the Control (100 ml/cmH2O compliance, open circles) and Experimental (50 ml/cmH2O compliance, filled circles) compartments of the model. (B) Ratio of aerosol deposition in the Experimental and Control compartment. 3 Hz IPV was delivered using the nebulizer construct shown in Figure 2A. ∗p < 0.05. [file Image_2.tif]

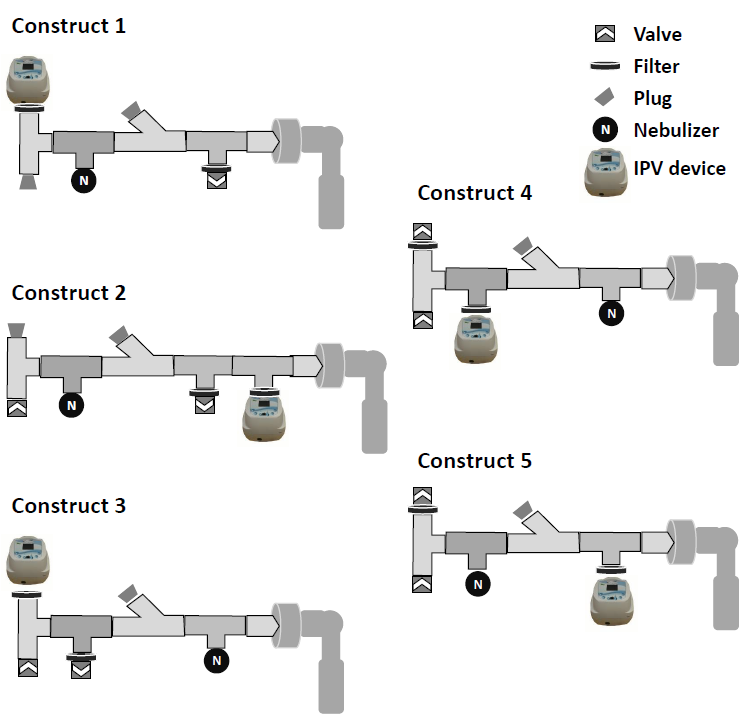

Supplement: FIGURE S3 — Nebulizer construct tested for IPV-enhancer aerosol deposition experiments. [file Image_3.tif]

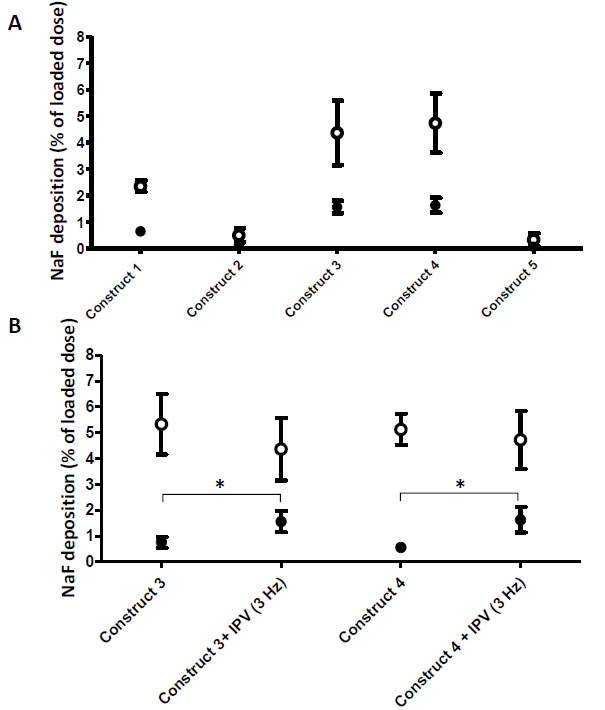

Supplement: FIGURE S4 — Aerosol deposition in the Control (100 ml/cmH2O compliance, open circles) and Experimental (50 ml/cmH2O compliance, filled circles) compartments of the model. (A) IPV (3 Hz, 40 cmH2O) was delivered during nebulization, using the 5 constructs shown in Supplementary Figure S3. (B) Constructs 3 and 4 were used either without or with IPV (3 Hz, 40 cmH2O). [file Image_4.tif]
